# Identification of plant-derived natural products as potential inhibitors of the *Mycobacterium tuberculosis* proteasome

**DOI:** 10.1186/1472-6882-14-400

**Published:** 2014-10-15

**Authors:** Yuejuan Zheng, Xin Jiang, Feng Gao, Junxiang Song, Jinxia Sun, Lixin Wang, Xiaoxia Sun, Zhenhui Lu, Huiyong Zhang

**Affiliations:** Department of Microbiology & Immunology, Shanghai University of Traditional Chinese Medicine, 1200 Cailun Road, Shanghai, 201203 China; Shanghai First People’s Hospital, Shanghai Jiao Tong University, Shanghai, 200080 the People’s Republic of China; Longhua Hospital, Shanghai University of Traditional Chinese Medicine, Shanghai, 200032 the People’s Republic of China

**Keywords:** *Mycobacterium tuberculosis*, Proteasome inhibitor, Plant-derived natural products, Flavonoids

## Abstract

**Background:**

The *Mycobacterium tuberculosis* (Mtb) proteasome has been established as a viable target for the development of anti-tuberculosis agents. In this study, the inhibitory activities of 100 plant-derived natural products on the Mtb proteasome were analyzed to identify novel potential inhibitors.

**Methods:**

The fluorescent substrate Suc-Leu-Leu-Val-Tyr-AMC can be hydrolyzed by the proteasome to release free AMC, the fluorescence of which is proportional to the proteasome activity. The inhibitory activities of 100 natural products (each at a final concentration of 200 *μ*M) were detected by this method using MG132 as a positive control.

**Results:**

Twelve of these natural products (10 of which were flavonoids) inhibited the activity of the Mtb proteasome by more than 65%. Comparison of the structural differences between the flavonoids with good inhibitory activity and those without inhibitory activity revealed that the hydroxyl at the flavonoid C ring C-3 or the hydroxyl/methoxyl at the flavonoid A ring C-6 were critical for the inhibition of proteasomal activity.

**Conclusions:**

These data indicate that flavonoids represent a basis for rational structural design in the process of novel anti-tuberculosis drug discovery.

## Background

*Mycobacterium tuberculosis* (Mtb) remains an important global pathogen that is considered to have infected one-third of the world’s population, causing the death of 1.3 million people, with 8.6 million new and relapsed infections in 2012 (World Health Organization Tuberculosis Data and Statistics, 2013) [1]. The development of new drugs is critical for the future control of tuberculosis (TB) and a number of promising compounds are currently in the pipeline at various stages of drug discovery and clinical development [2, 3]. New TB drugs are required to be active against both replicating and nonreplicating bacteria, and to penetrate tissues and granulomas to allow a reduced treatment duration [4].

The human immune system and classical antibacterial agents have the capacity to destroy Mtb in the proliferating state but not in the nonreplicating "drug tolerant" or "phenotypically drug resistant" state [5–7]. If the human immune system is compromised or medication is stopped, nonreplicating state Mtb quickly begin replicating [8]. The requirement for chemotherapy is prolonged for nonreplicating Mtb, which represents a major obstacle to the control of TB [9, 10]. Therefore, there is an urgent need to develop new drugs against nonreplicating Mtb to shorten the period of Mtb chemotherapy and to decrease the chances of treatment failure, Mtb relapse and the emergence of multidrug-resistant (MDR) strains [11, 12].

Mycobacteria are the only known bacterial pathogens with proteasomes are mycobacteria [13–15], which are essential for the degradation of certain proteins, survival of nitroxidative stress *in vitro* and maintenance of the nonreplicating state *in vivo* [16, 17]. Therefore, the Mtb proteasome may be required for virulence by mediating resistance to damage by reactive oxygen and nitrogen intermediates (ROI/RNI), and the acidic environment of the macrophage [18]. The Mtb proteasome removes proteins damaged by ROI or RNI, and shear detoxification proteins (such as repressors of genes that synthesize DNA repair enzymes). It also degrades transcription factors that regulate the expression of genes that counteract these host defenses [19, 20]. The Mtb proteasome has been established as a viable target for the development of anti-TB agents and proteasome inhibitors are implicated as potential drug candidates for the treatment of nonreplicating Mtb [21, 22]. In the current study, 100 natural products derived from plants were screened for inhibitory activity against the Mtb proteasome.

## Methods

### Plant-derived natural products and Mtb strain

One hundred natural products purified from plants were purchased from Shanghai Tauto Biotech (China) with purity >98% by HPLC. Suc-LLVY-7-amido-4-methylcoumarin (Suc-LLVY-AMC) was purchased from Boston Biochem (Cambridge, MA, USA). Stock solutions were prepared in DMSO.

Mtb H37Ra was cultivated on Middlebrook 7H9 medium (0.2% glycerol, 0.5% BSA, 0.2% dextrose, 0.085% NaCl and 0.05% Tween 80; pH 6.6) or on Middlebrook 7H11 plates.

### Screening of 100 natural products

Screening methods were performed as described previously with minor modifications [17, 23]. Mtb H37Ra mid-log phase (OD_580_ 1.0) cultures were centrifuged and the cell pellets were washed twice in phosphate buffered saline (PBS). The cell suspension was sonicated in a lysis buffer (50 mM Tris–HCl, pH 7.6, 50 mM NaCl, 5 mM MgCl_2_, 0.05% Triton X-100, 1 mM DTT and 0.5 mM EDTA). Lysates were centrifuged at 16,000 × *g* for 10 minutes and the supernatants were removed. The protein concentration of the supernatants was estimated with the Bradford assay. Proteasome activity of the supernatants was assessed. MG132 (a well-known proteasome inhibitor) was tested as a positive control. Reaction buffer containing substrate was added. Final concentrations were as follows: MG132 100 μM; test natural product 200 μM; Suc-LLVY-AMC 64 μM; protein concentration of Mtb lysates (supernatant) 25 μg/ml, HEPES 20 mM; EDTA 0.5 mM; SDS 0.34 mg/ml; pH 7.5. Each sample was tested in three duplicates. Plates were placed on an orbital shaker in an incubator at 37°C for 30 min and the fluorescence intensity of the free AMC was recorded using a luminescence microplate reader (Synergy-2, BioTek, USA) at excitation and emission wavelengths of 360 nm and 460 nm, respectively.

### IC_50_ assay

One hundred natural products were screened to identify those with an inhibitory activity exceeding 65%. The inhibitory activity was calculated as the concentration of inhibitor resulting in a percentage of reduction in fluorescent units (FU) compared to that of the control. The fluorescence intensity of the chosen products was analyzed using the above method with a series of different concentrations (400 μM, 200 μM, 100 μM, 50 μM, 25 μM, 12.5 μM and 6.25 μM) and the corresponding inhibition activities were calculated. The IC_50_ values of natural products with good inhibitory activities were calculated by dose response curve. The IC_50_ values were calculated by fitting with the four parameter logistic (4-PL) model, y = A2 + (A1-A2)/(1 + (×/IC_50_)^p), with OriginPro 8.1 (OriginLab, Inc.), where y is percent inhibition, x is inhibitor concentration, p is the slope of the concentration–response curve, A1 is the minimal inhibition ratio from three independent assays, and A2 is the maximal inhibition ratio from three independent assays.

## Results

### Inhibitory activities of 100 natural products

The one hundred selected natural products represent 12 categories including terpanoid (27), flavonoid (27), alkaloid (14), coumarin (8), quinone (6), phenol (5), organic acid (4), lignan (3), nucleoside (1), glycoside (2), steroid sapogenin (2), and stilbene (1).

**Figure 1:**
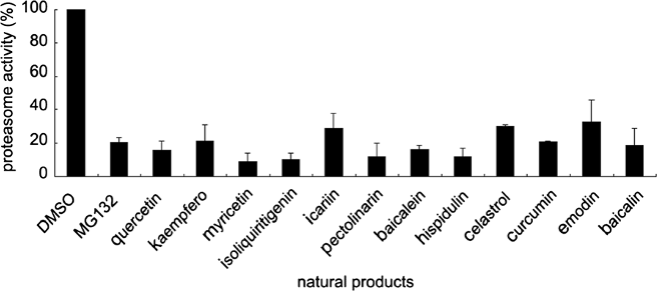
**Mtb proteasome inhibitory activities of 12 natural products (200 μM).** The inhibitory activities of 12 natural products (200 μM) were: hispidulin 82.06%, baicalein 84.30%, pectolinarin 88.69%, myricetin 84.50%, quercetin 74.40%, kaempferol 68.29%, isoliquiritigenin 83.60%, icariin 74.30%, baicalin 78.47%, curcumin 69.53%, celastrol 69.50% and emodin 73.09%, the inhibitory activity of MG132 (100 μM) is 79.66%. Data are shown as mean ± SD of three independent experiments.

The Mtb proteasome inhibitory activity of MG132 was 79.66% at 100 μM, and the proteasome inhibitory activities of 12 of the 100 natural products (at 200 μM) were more than 65%. Specifically, these 12 products were hispidulin, baicalein, pectolinarin, myricetin, quercetin, curcumin, kaempferol, isoliquiritigenin, icariin, baicalin, celastrol and emodin (Table 1 and Figure 1). In addition to emodin (quinones) and tripterine (terpenoids), the remaining 10 natural products belonged to the flavonoids group.

**Table 1:** **Mtb proteasome inhibitory activities of 100 natural products and chemical categories**

Categories	Name	IA	Name	IA	Name	IA	Name	IA
**Terpenoids**	tripterine^#^	69.56%	costunolide	41.75%	ginkgolide	39.90%	curcumol	34.66%
	dehydroandrographolide	32.67%	dehydrocostuslactone	32.15%	alantolactone	32.12%	evodine	31.77%
	tanshinone	25.63%	astragaloside A	23.59%	bilobalide	18.56%	swertiamain	14.39%
	artesunate	13.83%	betulinol	6.52%	ginsenoside	2.62%	limonin	1.61%
	glycyrrhetinic Acid	0.00%	hemslecin A	0.00%	madecassoside	0.00%	andrographolide	0.00%
	dipsacoside	0.00%	geniposide	0.00%	gentiopicroside	0.00%	tubeimoside A	0.00%
	notoginsenoside	0.00%	asiaticoside	0.00%	paclitaxel	0.00%		
**Flavonoids**	pectolinarin^#^	88.69%	myricetin^#^	84.50%	baicalein^#^	84.03%	isoliquiritigenin^#^	83.60%
	hispidulin^#^	82.06%	baicalin^#^	78.47%	quercetin^#^	74.40%	icarrin^#^	74.34%
	curcumin^#^	69.53%	kaempferol^#^	68.29%	alpha mangostin	64.16%	wogonoside	59.01%
	wogonin	53.97%	nevadensin	48.01%	genistein	45.74%	epigallocatechin gallate	45.21%
	mangiferin	31.88%	forsythin	20.02%	paeoniflorin	3.05%	tectoridin	0.00%
	luteolin	0.00%	rutin	0.00%	silmyarin	0.00%	puerarin	0.00%
	apigenin	0.00%	daidzein	0.00%	diosmetin	0.00%		
**Alkaloids**	indirubin	45.99%	martin hydrochloride	43.66%	matrine	23.53%	sophoridine	11.85%
	sinomenine	3.77%	bergenin	3.67%	peimine	3.05%	berberine	0.00%
	lycorine hydrochloride	0.00%	hanfangchin B	0.00%	hanfangchin A	0.00%	camptothecin	0.00%
	strychnine	0.00%	cepharanthine	0.00%				
**Coumarins**	imperatorin	15.90%	scoparone	0.00%	praeruptorin A	0.00%	umbelliferone	0.00%
	coumarin	0.00%	daphnetin	0.00%	fraxin	0.00%	osthole	0.00%
**Quinones**	emodin^#^	73.09%	alkannin	52.36%	rubimaillin	46.37%	1,8-dihydroxyanthraquinone	36.30%
	aloe-emodin	34.30%	alizarin	0.00%				
**Phenols**	gallogen	62.50%	salidroside	34.04%	paeonol	0.00%	resveratrol	0.00%
	salicin	0.00%						
**Organic acid**	diffractic acid	0.00%	syringic acid	0.00%	shikimicacid	0.00%	chlorogenic acid	0.00%
**Lignans**	arctigenin	27.10%	deoxyschizandrin	23.86%	magnolol	0.00%		
**Nucleoside**	cordycepin	7.77%						
**Glycoside**	amygdalin	1.87%	2,3,5,4′-tetrahydroxy stilbene-2-O-β-D-glucoside	0.00%				
**Stilbenes**	polydatin	0.00%						
**Steroid sapogenin**	sarsasapogenin	0.00%	diosgenin	0.00%				
**Positive control**	MG132	79.66%						

IA: inhibitory activity; ^#^natural products with inhibitory activity of Mtb proteasome more than 65%. Different categories of natural products were highlighted using bold texts in the table. The concentration of natural products was 200 μM with the exception of MG132 (100 μM). Data are shown as mean of three independent experiments.

### IC_50_ values of 12 natural products against the Mtb proteasome

**Figure 2:**
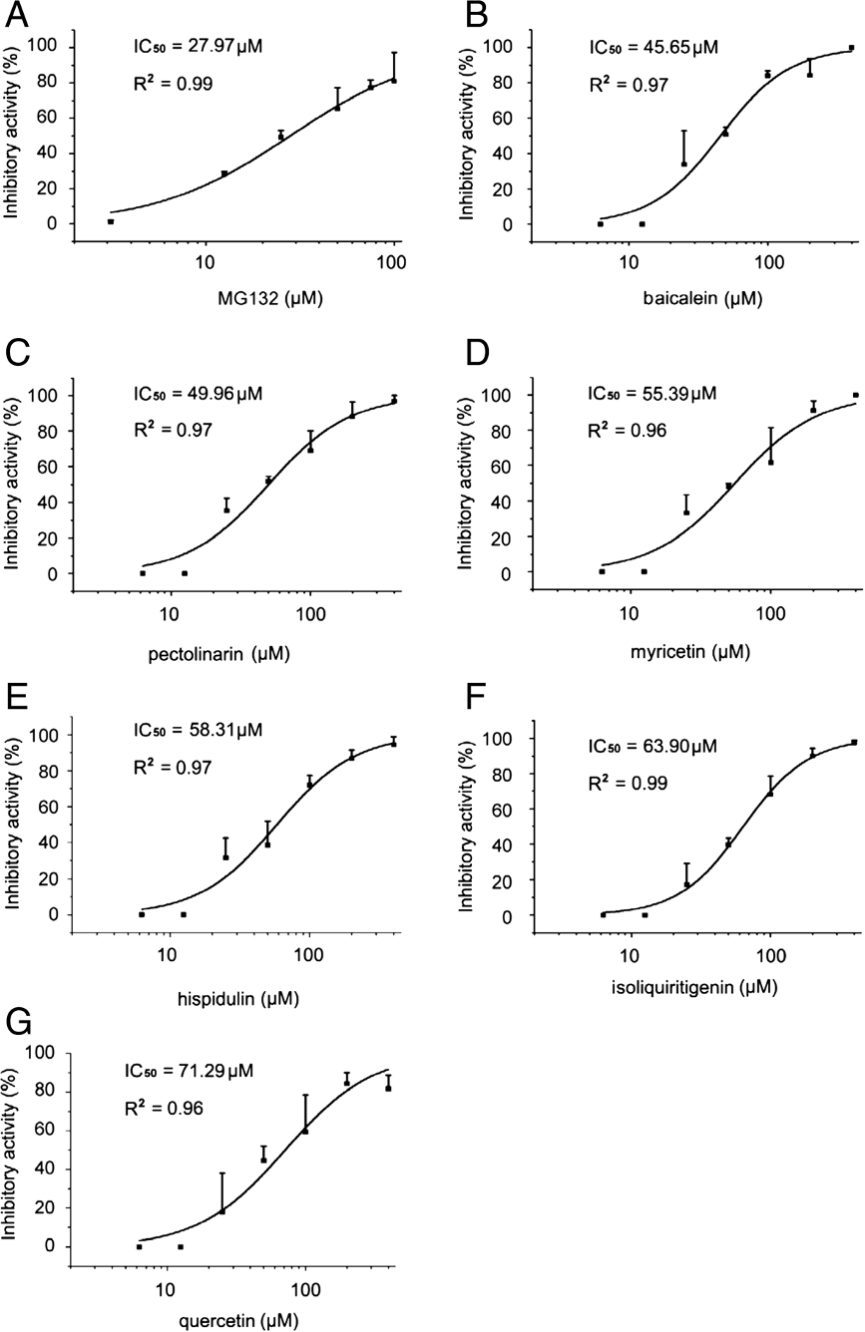
**Dose response curves of inhibitory activities of MG132, baicalein, pectolinarin, myricetin, hispidulin, isoliquiritigenin and quercetin.** The IC_50_ values were calculated by fitting with the four parameter logistic (4-PL) model: **A**. MG132 IC_50_=27.97 μM, R^2^=0.99. **B**. baicalein IC_50_=45.65 μM, R^2^=0.97. **C**. pectolinarin IC_50_=49.96 μM, R^2^=0.97. **D**. myricetin IC_50_=55.39 μM, R^2^=0.96. **E**. hispidulin IC_50_=58.31 μM, R^2^=0.97. **F**. isoliquiritigenin IC_50_=63.90 μM, R^2^=0.99. **G**. quercetin IC_50_=71.29 μM, R^2^=0.96. R^2^, adjust R square values. Dose response curves of inhibitory activity were presented. Data are shown as mean ± SD of three independent experiments.

**Figure 3:**
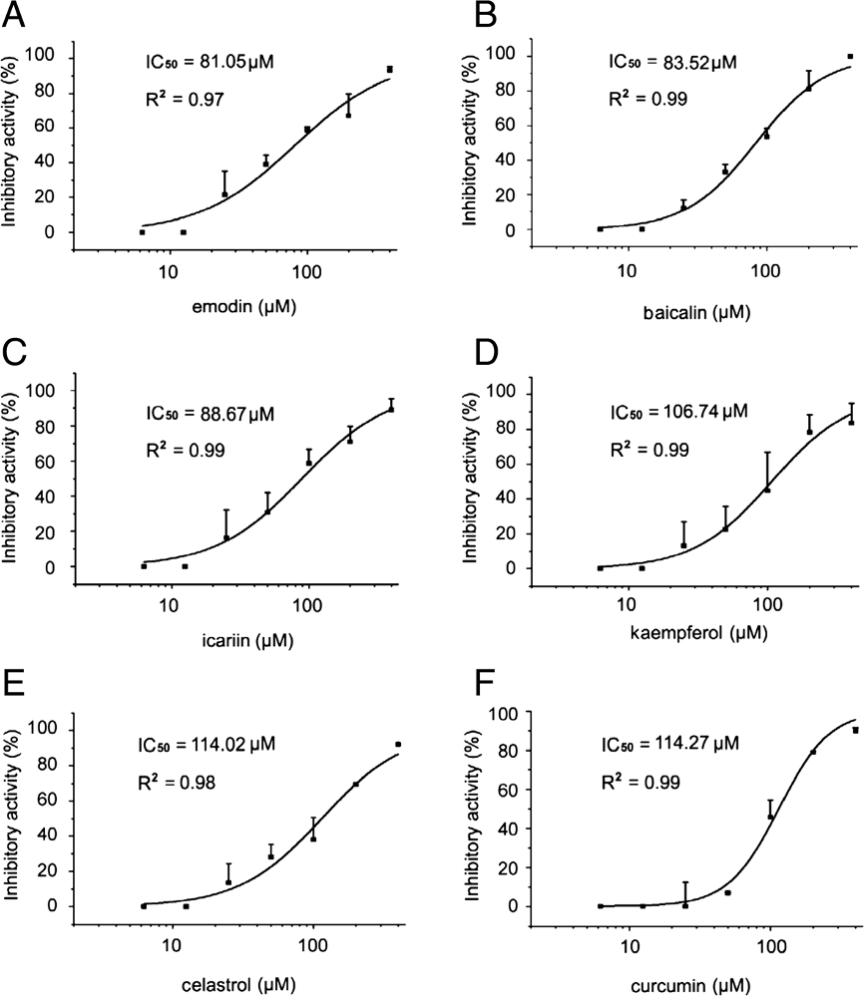
**Dose response curves of inhibitory activities of emodin, baicalin, icariin, kaempferol, celastrol and curcumin.** The IC_50_ values were calculated by fitting with the four parameter logistic (4-PL) model: **A**. emodin IC_50_=81.05 μM, R^2^=0.97. **B**. baicalin IC_50_=83.52 μM, R^2^=0.99. **C**. icariin IC_50_=88.67 μM, R^2^=0.99. **D**. kaempferol IC_50_=106.74 μM, R^2^=0.99. **E**. celastrol IC_50_=114.02 μM, R^2^=0.98. **F**. curcumin IC_50_=114.27 μM, R^2^=0.99. R^2^, adjust R square values. Dose response curves of inhibitory activity were presented. Data are shown as mean ± SD of three independent experiments.

Twelve natural products showed concentration-dependent proteasomal inhibitory activities. IC_50_ values were calculated by fitting with the four parameter logistic (4-PL) model (Figures 2, 3 and Table 2) and the minimal IC_50_ of the 12 natural products was 45.65 μM.

**Table 2:** **IC**_**50**_ **of 12 natural products**

Natural products	IC _50_ (μM)	Natural products	IC _50_ (μM)
baicalein	45.65	emodin	81.05
pectolinarin	49.96	icariin	88.67
quercetin	71.29	kaempfero	106.74
hispidulin	58.31	baicalin	83.52
myricetin	55.39	celastrol	114.02
isoliquiritigenin	63.90	curcumin	114.27
MG 132(control)	27.97		

The IC_50_ values of 12 natural products were calculated by dose response curve.

## Discussion

Natural products, as similar as secondary metabolites, exhibit diversity in structures and biological activities. These compounds play an important role in the discovery of lead compounds and more than 50% of FDA-approved drugs are either natural products or natural product derivatives [24, 25]. Furthermore, natural products have specific selectivity for cellular targets [26], with biologically active natural products providing selective ligands for disease-related targets [27]. The *in vitro* inhibitory activity of crude extracts and/or pure active compounds extracted from plants against Mtb has been extensively reported [28–31].

Twelve of the 100 natural products selected for investigation in this study exhibited inhibitory activities against the proteasome exceeding 65%. Among these, 10 natural products were flavonoids. Thus, in our study, flavonoids showed better inhibitory activities against the Mtb proteasome than other categories, implicating flavonoids as potential proteasome inhibitors. Although some flavonoids showed inhibitory activities against the Mtb proteasome, the lowest IC_50_ (baicalein, 45.65 μM) was relatively higher than that of the positive control (MG132, 27.97 μM). Our study provides an insight into the unique features of potential proteasome inhibitors.

**Figure 4:**
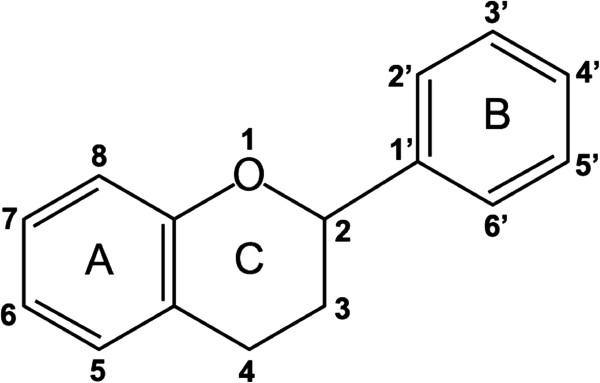
**Basic structures of flavonoids.**

**Figure 5:**
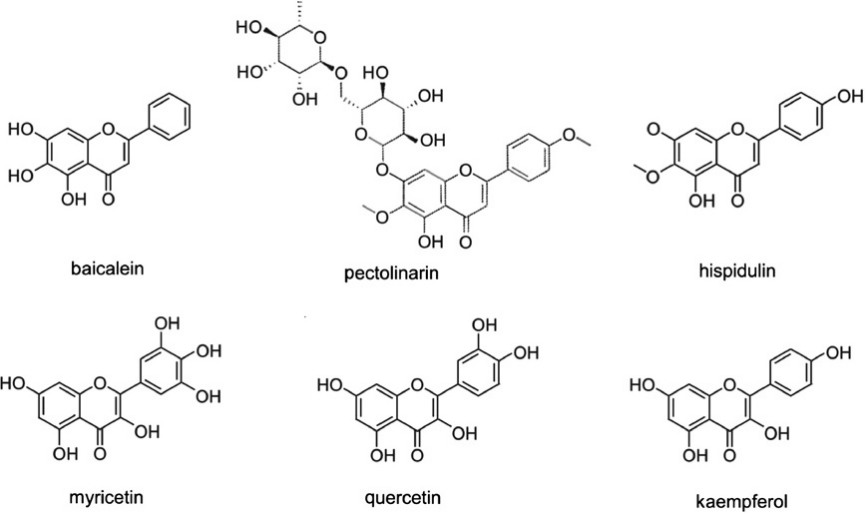
**Six natural products of the flavonoids with good inhibitory activity on the Mtb proteasome.** Inhibitory activities of six flavonoids (each 200 μM) were as follows: baicalein 84.30%, pectolinarin 88.69%, hispidulin 82.06%, myricetin 84.50%, quercetin 74.40% and kaempferol 68.29%. The common structural features are the hydroxyl at the C ring C-3 (myricetin, quercetin and kaempferol), or the hydroxyl (baicalein) or the methoxyl (pectolinarin and hispidulin) at the A ring C-6.

**Figure 6:**
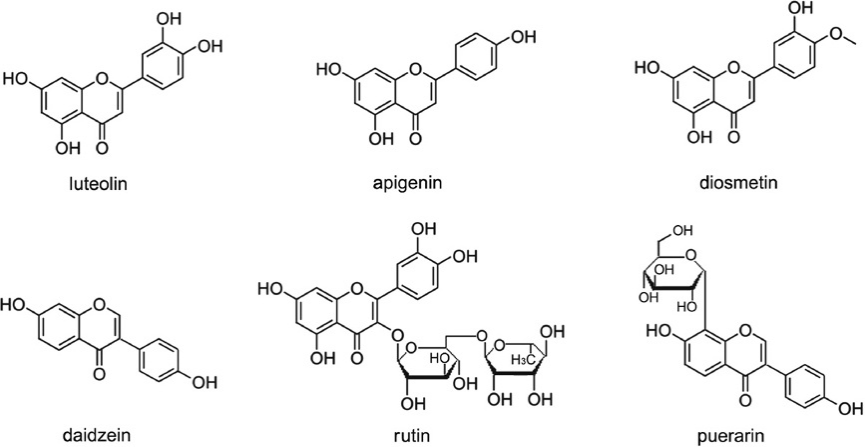
**Six natural products of the flavonoids with little inhibitory activity on the Mtb proteasome.** Inhibitory activities of six flavonoids (at 200 μM) were zero. Compared with Figure 5, these compounds are without the hydroxyl at the C ring C-3 and the hydroxyl or methoxyl at the A ring C-6.

Natural flavonoids, which exist widely in nature, are secondary metabolites of plants and perform a wide range of functions. The chemical structure of flavonoids is based on a fifteen-carbon skeleton consisting of two benzene rings (A and B) linked by a heterocyclic pyrane ring (C), showing a common three ring structure (C_6_–C_3_–C_6_) (Figure 4) [32]. In this study, despite the finding that the inhibitory activities of flavonoids against the Mtb proteasome was better than that of other categories, there was a wide variation in the inhibitory activities between different flavonoid products. The structures of flavonoids with good inhibitory activity were shown in Figure 5 and those with little inhibitory activity were shown in Figure 6. The common structures of the flavonoids with effective inhibitory activity against the Mtb proteasome are hydroxyl residue at the C ring C-3, or hydroxyl/methoxyl residue at the A ring C-6. This indicates some key functional structures of flavonoids with inhibitory activity against the Mtb proteasome.

The well-established free-radical scavenging ability of flavonoids depends on electron-donating hydroxyl group substitutions of the aromatic A ring and the heterocyclic C ring [33]. The C-2–C-3 double-bond conjugated to a C-4 carbonyl group on the C ring is responsible for antioxidant activity [23]. Comprehensive analysis of the correlations between functional groups in the flavonoid structures and their proteasome inhibitory activities are necessary to evaluate the effects of modifications at the C ring C-3 and the A ring C-6.

## Conclusions

The flavonoid structure has been shown to be a viable template for the design of potent Mtb proteasome inhibitors. This information forms the basis of the rational design of flavonoid analogs retaining the key functional groups, which will focus direct screening and will be helpful to accelerate the discovery of potent inhibitors of the Mtb proteasome.
